# Appropriate statistical methods for analysing partially nested randomised controlled trials with continuous outcomes: a simulation study

**DOI:** 10.1186/s12874-018-0559-x

**Published:** 2018-10-11

**Authors:** Jane Candlish, M. Dawn Teare, Munyaradzi Dimairo, Laura Flight, Laura Mandefield, Stephen J. Walters

**Affiliations:** School of Health and Related Research (ScHARR), University of Sheffield, 30 Regent Street, S1 4DA, Sheffield, UK

**Keywords:** Clustering, Randomised controlled trial, Partially nested, Partially clustered, Therapist effects, Individually randomised group treatment, Individually randomised cluster trial, Intervention studies

## Abstract

**Background:**

In individually randomised trials we might expect interventions delivered in groups or by care providers to result in clustering of outcomes for participants treated in the same group or by the same care provider. In partially nested randomised controlled trials (pnRCTs) this clustering only occurs in one trial arm, commonly the intervention arm. It is important to measure and account for between-cluster variability in trial design and analysis. We compare analysis approaches for pnRCTs with continuous outcomes, investigating the impact on statistical inference of cluster sizes, coding of the non-clustered arm, intracluster correlation coefficient (ICCs), and differential variance between intervention and control arm, and provide recommendations for analysis.

**Methods:**

We performed a simulation study assessing the performance of six analysis approaches for a two-arm pnRCT with a continuous outcome. These include: linear regression model; fully clustered mixed-effects model with singleton clusters in control arm; fully clustered mixed-effects model with one large cluster in control arm; fully clustered mixed-effects model with pseudo clusters in control arm; partially nested homoscedastic mixed effects model, and partially nested heteroscedastic mixed effects model. We varied the cluster size, number of clusters, ICC, and individual variance between the two trial arms.

**Results:**

All models provided unbiased intervention effect estimates. In the partially nested mixed-effects models, methods for classifying the non-clustered control arm had negligible impact. Failure to account for even small ICCs resulted in inflated Type I error rates and over-coverage of confidence intervals. Fully clustered mixed effects models provided poor control of the Type I error rates and biased ICC estimates. The heteroscedastic partially nested mixed-effects model maintained relatively good control of Type I error rates, unbiased ICC estimation, and did not noticeably reduce power even with homoscedastic individual variances across arms.

**Conclusions:**

In general, we recommend the use of a heteroscedastic partially nested mixed-effects model, which models the clustering in only one arm, for continuous outcomes similar to those generated under the scenarios of our simulations study. However, with few clusters (3–6), small cluster sizes (5–10), and small ICC (≤0.05) this model underestimates Type I error rates and there is no optimal model.

**Electronic supplementary material:**

The online version of this article (10.1186/s12874-018-0559-x) contains supplementary material, which is available to authorized users.

## Background

Randomised controlled trials (RCTs) are often categorised into two types: individually randomised controlled trials (iRCTs) where participants are individually randomised to treatment arms to receive one of the investigative treatments; and cluster randomised controlled trials (cRCTs) where groups of participants (clusters) are randomised to treatment arms. We may expect outcomes for participants within the same cluster to be more similar than those from different clusters. The similarity can arise due to participants in the same cluster receiving care from the same health provider or interacting with one another [1]. The implications of clustering in cRCTs are widely acknowledged [1, 2]. Clustering results in a reduction in statistical efficiency in cRCTs and if ignored standard errors and *p*-values for intervention effects are typically underestimated.

Clustering can also occur in iRCTs. For instance, clustering of participants outcomes due to receiving treatment as part of a group-based parenting intervention [3], treatment in specialist clinics for the treatment of venous leg ulcers [4], or participants under the care of a surgeon for comparison for hemostasis in elective benign thyroid surgery [5]. The care provider or group dynamics may play a role in the causal pathway of the intervention effect. We might expect correlated outcomes between individuals either in the same group or receiving treatment from the same care provider.

Standard sample size and analysis methods for iRCTs rely on the assumption of independence between participants, which is violated when clustering is present. The ‘clustering effect’ is commonly quantified using the intracluster correlation coefficient (ICC). The ICC measures the extent to which outcomes from participants within the same cluster are correlated to one another [1]. When designing and analysing iRCTs with clustering we need to consider implications of the potential lack of independence. Ignoring clustering in the analysis can lead to over precise results and consequently incorrect conclusions [1]. Clustering of any form results in a reduction in the effective sample size, hence, there is a reduction in the power to detect an intervention effect if it truly exists.

In addition to obtaining sufficient power and accurate results, accounting for clustering enables us to estimate the ICC. ICCs are often important for the interpretation of trial results; we may be directly interested in the intervention group or care provider effects. ICCs are also key when calculating sample sizes for RCTs with clustering, in order to maintain power [1].

An increasingly applied design in healthcare and education research is a partially nested randomised controlled trial (pnRCT) [6], where participants are individually randomised to trial arms and clustering of outcomes occurs in only one arm of the trial [7] (also termed partially clustered trials). The STEPWISE trial is an example of a pnRCT, assessing a structured lifestyle education programme aimed at supporting weight loss for adults with schizophrenia and first episode psychosis in a community mental health setting. Individuals were randomised to either an intervention arm of group-based lifestyle education sessions or a control arm receiving usual care at the individual level [8].

Specific statistical methods need to be used for analysing pnRCTs. Consequently, there has been a considerable growth in the methodology literature (particularly in the fields of psychotherapy and educational research) in the past few decades both proposing and reviewing statistical methods for pnRCTs.

Table 1 presents a summary of relevant literature on the analysis of pnRCTs. This expands on the literature search by Flight et al. [9] summarising models for the analysis of pnRCTs. Sample size calculations for pnRCTs have been addressed elsewhere [10–14]. Analysis methods for pnRCTs have mainly focussed on using mixed-effects models, individual-level models which account for the hierarchical structure of the data [6, 7, 9, 15–19]. These models allow us to control for baseline covariates and represent the different levels in the data, including cluster, individual, and repeated measures (where applicable). In addition to accounting for the clustering, we may expect the variance of the individual errors to differ between trial arms in pnRCTs, termed heteroscedastic variance [7]. When a clustered intervention arm is compared to a non-clustered control arm the between-cluster variation in the intervention arm is not present in the control arm. The clustered intervention may result in a decrease or increase of the individual level variability.

**Table 1:** Summary of relevant literature on analysis of pnRCTs

Paper	Relevant themes	Range of values^a^	Findings
Schweig & Pane [16]	Describe and compare models for pnRCTs with non-compliance using a simulation study.	Simulation for two levels of clustering, exact cluster sizes (*m*) unclear in paper, *c*_*school*_ = 37, *c*_*class*_ = 177, λ_*B*_ = 2, 8, *ρ*_*school*_ = 0.005, 0.05, 0.15, *ρ*_*class*_ = 0.0004, 0.10, 0.25, and θ = 0.087.	Clustering and non-compliance may have a substantial impact on statistical inference about intention-to-treat effects. Provide methods that may accommodate pnRCT with non-compliance, recommend using complier average causal effect estimate (CACE) and scaling by the proportion of compliers. No mention of degrees of freedom, we have assumed they used default degrees of freedom method available in R lme packages.
Flight et al. [9]	Compare models applied to four examples of pnRCTs. Compare three different methods for classifying the non-clustered control arm in pnRCTs, including: singleton clusters, one large cluster and pseudo clusters.	Examples with {*m*, *c*} = {36, 8; 24, 7; 14, 8; 5, 6}, and estimated *ρ* = < 0.0001, 0.02, 0.007.	Recommend use of the heteroscedastic model, recommendations based only on re-analysis of case studies. Methods for classifying the non-clustered control arm in pnRCTs had a large impact in fully clustered mixed effects models and no measurable impact in partially nested mixed-effects models. ICCs in four examples were small.
Sterba [27]	Review of modelling developments for pnRCTs, focused on those particularly relevant to psychotherapy trials.		Recommend the inclusion of cluster variability in analysis model as it provides insight into treatment process (rather than treating it as a nuisance). Annotated Mplus commands for models
Lohr, Schochet & Sanders [19]	Report presenting a guide to design and analysis issues for pnRCTs in education research, using example trials. Discussion of degrees of freedom issue in Appendix.		Guidance document, defines pnRCT in context of education research and show methods to analyse these using SAS. Provide SAS commands for model fitting in examples.
Korendijk [18]	Compare models for pnRCTs using simulation study, investigate mis-specification for the estimation of the parameters and their standard errors.	Simulation study with *m* = 5, *c* = 10, 30, 50, 100, *ρ* = 0.05, 0.1, 0.2, λ_*A*_ = 1, *d* = 0.3.	All models perform comparably with respect to fixed effect estimates. Recommend use of partially nested mixed-effects model. Simulations were under null and ICC always greater than zero. No mention of degrees of freedom, we have we assumed default degrees of freedom used from MLwiN software, and homoscedasticity was assumed for ndividual variances between the two arms.
Sanders [17]	Compare models for pnRCTs using simulation study in terms of Type I error and power	Simulation study with {*m*, *c*} = {2, 10; 4, 4; 5, 4; 10, 2}, *ρ* = 0, 0.1, 0.2, 0.3, 0.4, 0.5, λ_*A*_ = 1, and ω^2^ = 0, 0.01, 0.059, 0.138.	Type I error rate increased as ICC increased, Satterthwaite degrees of freedom had better control than Kenward-Roger degrees of freedom. Found using mixed-effects model for pnRCT when ICC is zero likely leads to never detecting intervention effects, observed Type I error rates nearly non-existent under all scenarios with ICC equal to zero. Recommend to evaluate if ICC is significantly different from zero prior to selecting analysis method. Homoscedasticity was assumed for individual variances between the two arms.
Baldwin et al. [15]	Compare analysis models for pnRCT simulation study, comparing three degrees of freedom calculations, and a pnRCT example.	Simulation for *m* = 5, 15, 30, *c* = 2, 4, 8, 16, *ρ* = 0, 0.05, 0.1, 0.15, 0.3, λ_*B*_ = 0.25, 1, 4, and *d* = 0, 0.5.	Recommend pnRCTs take account of heteroscedasticity. Satterthwaite and Kenward-Roger degrees of freedom control Type I error rate. The heteroscedastic model provides an unbiased estimate and little reduction in power compared to the homoscedastic model. Argue that using a partially nested mixed-effects model only a problem for statistical inference when the number of clusters is small. The number of clusters has greater impact on power in pnRCTs. At least eight, preferably 16 clusters, to maintain Type I error rate.
Bauer et al. [6]	Review of RCTs to ascertain the prevalence of pnRCTS in four public health and clinical research journals. Analysis models for pnRCTs extended to include pre-test measures as covariates, individual and group level covariates, and example of pnRCT	Example with clustering in one arm *c* = 41, *m* = 9, and estimated *ρ* = 0.02.	Out of 94 RCTs, 32% were pnRCTs, 40% iRCTs and 27% cRCT. None used methods specific to pnRCTs. Example pnRCT data could be analysed using mixed-effects models. Argue pnRCTs “often increase external validity at the expense of internal validity” (p.20).
Roberts & Roberts [7]	Examine the case of pnRCTs, heterogeneity, comparison of analysis methods for simulation study and present an example.	Simulation for m = 6, *c* = 8, *ρ* = 0, 0.1, 0.2, 0.3, λ_*A*_ = 0.5, 0.75, 1, 1.25, 1.5, 1.75, 2 and *d* = 0.	Recommend pnRCTs take account of heteroscedasticity. Satterthwaite unequal variances t-test gave robust to heteroscedasticity. The heteroscedastic model gives slightly inflated test size for large ρ: suggest Satterthwaite degrees of freedom as a solution.
Lee & Thompson [28]	Describe analysis models for iRCTs with clustering and apply to two examples (using Bayesian approach)		Show that ignoring clustering may underestimate uncertainty, leading to incorrect conclusions.
Hoover [34]	Statistical tests for RCTs with clustering that differ across trial arms.	Example with clustering in both arms with *m* = 7 − 12, *c* = 3.	Provide an adjustment for the independent samples t-test for pnRCTs. Statistical impact of heterogeneity effect increases as the cluster size increases, and as heterogeneity increases. The test does not allow for the inclusion of covariates, multiple treatments, baseline measures, or non-normally distributed outcomes.

^a^*m* = cluster size, *c* = number of clusters, *ρ*= ICC, *d* = standardised effect size, ω^2^= Omega Squared effect size percent of variability accounted for by treatment condition, λ_*A*_= ratio of total variance in control arm compared to clustered, λ_*B*_= ratio of individual variance in control arm compared to clustered arm. Ordered by year of publication

In this study, we use a series of simulations to evaluate the statistical analysis models for two-arm parallel pnRCTs with continuous outcomes, assessing a range of scenarios including the effect of cluster size and the number of clusters. In theory, the mixed-effects models can be formulated so that they do not model clustering in the control arm, however, when running these models in statistical software packages it is necessary to impose some form of clustering in the control arm. The literature identified in Table 1 highlighted that research to date is lacking in addressing the best way to treat the non-clustered control arm when fitting the models in statistical software, using scenarios of relevance in the field of public health with small clusters and small ICCs [9], and evaluating the effect of the variance ratio of the residuals on the model fit. In pnRCTs we may have small numbers of clusters [9], thus we evaluate the impact of the number of clusters on statistical inference and if statistical inference remains valid using mixed-effects models.

We evaluate and provide recommendations for the most appropriate analysis methods for pnRCTs, including:

1. where mixed-effects models are necessary,
2. methods of specifying the clusters in the non-clustered arm when fitting a model and the impact these have on the analysis,
3. the impact of cluster sizes and the number of clusters on statistical inference and,
4. the impact of heteroscedastic individual variance between trial arms on statistical inference.

## Methods

### Methods for analysis of partially nested trials

In this section, we present the main modelling approaches currently available and used for pnRCTs, including ignoring clustering altogether, imposing clustering in the non-clustered control arm, and explicitly modelling the partially nested design by modelling clustering only in the intervention arm.

It is possible to account for clustering by including each cluster as a fixed effect in a standard regression model, in addition to a fixed effect representing the intervention effect. Although this method is simple to implement, it is not recommended. Firstly, it does not reflect the study design of a pnRCT and may require a large number of fixed effects to be fitted lowering the degrees of freedom [9]. Secondly, if clusters are of size one there is insufficient information to estimate both the intervention effect and the cluster effect for each cluster. Finally, it will generally underestimate the intervention effect variability as the cluster level variability is removed.

Table 2 presents a summary of the models for the analysis of pnRCTs using findings from the literature search by Flight et al. [9]. We define: *y* as a continuous outcome, *i* is the individual participant indicator, *j* is the cluster indicator, *t* is the intervention indicator (0 = control, 1 = intervention), *θ* is the intervention effect, *β*_0_ is an intercept term. Error terms are defined depending on the model procedure, represented using *ϵ*, *u*, and *r*, where *u* represents the between cluster variation and *ϵ* and *r* represent individual level variation.

**Table 2:** Models for the analysis of pnRCTs

Model description		Statistical model	Heteroscedastic residuals
Model 1	Linear regression (ignore clustering)	*y*_*i*_ = *β*_0_ + *θt*_*i*_ + *ϵ*_*i*_ • ${\epsilon}_i\sim N\left(0,{\sigma}_{\epsilon}^2\right)$ the individual level variation	No
Model 2	Fully clustered (impose clustering)	*y*_*ij*_ = *β*_0_ + *θt*_*ij*_ + *u*_*j*_ + *ϵ*_*ij*_ • ${u}_j\sim N\left(0,{\sigma}_u^2\right)$ a random effects term representing between cluster variation • ${\epsilon}_{ij}\sim N\left(0,{\sigma}_{\epsilon}^2\right)$ the individual level variation	No
Model 3	Partially nested homoscedastic	*y*_*ij*_ = *β*_0_ + *θt*_*ij*_ + *u*_*j*_*t*_*ij*_ + *ϵ*_*ij*_ • ${u}_j\sim N\left(0,{\sigma}_u^2\right)$ a random effects term representing between-cluster variation in clustered arm • ${\epsilon}_{ij}\sim N\left(0,{\sigma}_{\epsilon}^2\right)$ the individual level variation	No
Model 4	Partially nested heteroscedastic	*y*_*ij*_ = *β*_0_ + *θt*_*ij*_ + *u*_*j*_*t*_*ij*_ + *r*_*ij*_(1 − *t*_*ij*_) + *ϵ*_*ij*_*t*_*ij*_ • ${u}_j\sim N\left(0,{\sigma}_u^2\right)$ a random effects term representing between cluster-variation in clustered arm • ${r}_{ij}\sim N\left(0,{\sigma}_r^2\right)$ the individual level variation in the non-clustered control arm. • ${\epsilon}_{ij}\sim N\left(0,{\sigma}_{\epsilon}^2\right)$ the individual level variation in the clustered arm	Yes

Model 1 (Table 2) is the linear regression model which ignores the clustering and uses analysis for non-clustered trials, assuming independence between individuals regardless of whether they are in the same cluster. This infers that the conditional variance of *y* in both the intervention and control arms is equal. If the outcomes of individuals in the same cluster are correlated, the independence assumption is violated and we underestimate uncertainty around intervention effects when using the linear regression model above.

Model 2 (Table 2) is the fully clustered mixed-effects model which includes the cluster as a random effect; this includes variability at both the individual and cluster level. The mixed-effects model with imposed clustering of the control arm requires the estimation of a random cluster effect for both intervention and control groups. Some options for the imposed clustering in the control arm are given in Table 3. The variance of the control arm and intervention arm are assumed to be the same (homoscedastic). When the variance is believed to differ between control and intervention arm model 2 is not appropriate as it does not account for heteroscedasticity. Models 3 and 4 (Table 2) apply the cluster effect to the clustered arm only [7, 10, 11, 14], we term these the partially nested models.

**Table 3:** Options for imposing clustering in the non-clustered control arm

Option	Cluster	Intervention
1	*j* = 0	*j* = 1, …, *c*
2	*j* = 1, …, *l*	*j* = *l* + 1, …, *c*
3	*j* = 1, …, *k*	*j* = *k* + 1, …, *c*

Individuals in the non-clustered control arm are assumed independent. This accurately reflects the design of the study with clustering only in one arm. In the partially nested homoscedastic model, we apply the random effect *u*_*j*_ to the clustered intervention arm only; between-cluster variability is only present for the intervention arm. Model 3 is homoscedastic as the variance of the individual errors, *ϵ*_*ij*_, between arms is the same. In practice, the variance of the individual errors may differ between trial arms [7]. Therefore, model 3 is extended to a partially nested heteroscedastic model, model 4, this allows for differing individual errors between intervention and control arms but does not constrain the form of heteroscedasticity.

### Imposed clustering in the control arm

Regardless of whether or not the model assumes clustering in one (models 3 and 4) or both arms (model 2), within the statistical software package a decision must be made about how to code the cluster indicators in the control arm. One method is to impose clusters for all individuals, including those in the control arm, and use analysis for cRCTs with clustering in both arms.

Table 3 represents the different options for imposing clustering, *j*, in the control arm, *l* is the number of individuals in the control arm and *k* is the number of arbitrary clusters in the control group. Option one treats the control group as one single cluster; option two treats each individual in the control arm as their own cluster of size one (singleton clusters) giving *j=l* clusters in the control arm. ICC estimation can be problematic with options one and two, in theory, it is not possible to estimate between-cluster variability in option one, or estimate within cluster variability in the control group using option two [20]. Option three imposes artificial pseudo-random clusters in the control group to overcome the problem of estimating within or between-cluster variability. The number of arbitrary clusters, *k*, needs to be considered. We chose it to be equal across treatment arms. In addition, option three will likely result in a lower ICC estimation due to the assumed independence of control participants.

In our simulation study, the fully clustered model 2 is parametrised using the imposed clustering from Table 3. The models are:

- Model 2.1 fully clustered mixed-effects model with singleton clusters in the control arm;
- Model 2.2 fully clustered mixed-effects model with one large cluster in the control arm;
- Model 2.3 fully clustered mixed-effects model with pseudo clusters in the control arm.

Flight et al. [9] investigated the effect of the different methods of imposing clustering in the control arm presented in Table 3 in four pnRCT case-studies. The four case-studies covered trials evaluating the effect of: specialist leg ulcer clinics (clustered by clinic), acupuncture for low back pain (clustered by acupuncturist), postnatal support in the community (clustered by community support worker), and telephone befriending for maintaining quality of life in older people (clustered by volunteer facilitator). Little difference was found between the different methods for the fully clustered mixed-effects models and there was no difference between the different methods for the partially nested mixed-effects models.

We anticipated that the method of imposing the clustering in the control arm does not affect the results of the methods which model clustering in only one arm, however, this evaluated in the simulation study.

### Degrees of freedom for fixed effect estimates

In the mixed-effects models above we wish to carry out significance tests for the intervention effect. In addition to the correct choice of model, the test statistics and degrees of freedom in mixed-effects models also need to be considered. For large sample sizes in mixed-effects models, the test statistics for fixed effects can be assumed Normally distributed. However, for small samples, the t-distribution is generally used as an approximation of the distribution of the test statistic. Estimating the degrees of freedom for the t-distribution is unclear for pnRCTs and will affect both the significance test and the confidence intervals of the intervention effect estimate.

Comparison of degrees of freedom correction methods has been undertaken for cRCTs and pnRCTs with small numbers of clusters [15, 21]. The Satterthwaite small-sample degrees of freedom correction takes into account the variance structure of the data, for pnRCTs, it has been shown to be superior to the between-within method for maintaining Type I error rates (and comparable to the Kenward-Roger method) [15]. Following these results, the Satterthwaite approximation was used to calculate degrees of freedom (using dfmethod() option for mixed, available in Stata 14 onwards [22]).

### Simulation study

#### Overview

We performed a simulation study to evaluate the statistical analysis models for pnRCTs presented in Table 2, and the imposed clustering of the control arm in Table 3 [23]. All models were fitted using a restricted maximum likelihood procedure (REML). All simulations were done in Stata [22], graphs produced using ggplot2 [24] in R [25]. See Additional file 1 for simulation code.

#### Data-generating mechanism

We simulated data to replicate a two-arm parallel pnRCT trial with a non-clustered control arm and a clustered intervention arm (randomised 1:1) and a continuous outcome. We simulated data under various design scenarios and under both the null (*θ* = 0) and alternative hypothesis (*θ* = *A*, where *A* ≠ 0).

Data were simulated from the following model with the intercept set to zero and group allocation denoted by *t* (*t* = 0 for control, *t* = 1 for intervention arm):

- For the intervention arm $\left(t=1\right)\kern0.28em {y}_{ij}=\theta +{u}_j\sqrt{\rho }+{z}_{ij}\sqrt{1-\rho }$
- For the control arm $\left(t=0\right)\kern0.28em {y}_{ij}={z}_{ij}\sqrt{\gamma \left(1-\rho \right)}$

**Fig. 1:**
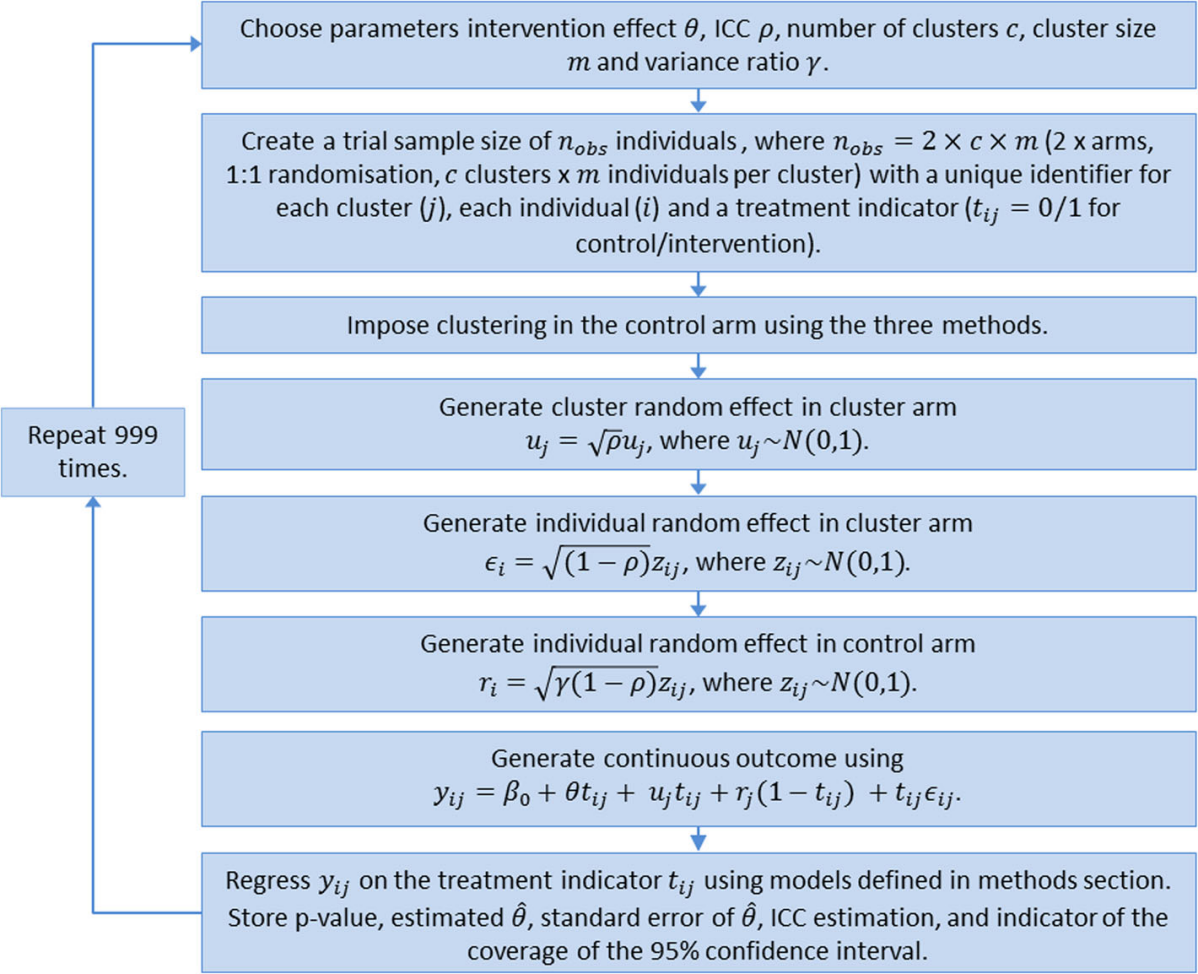
Flowchart representing the simulation study steps

Where *u*_*j*_~*N*(0, 1) and *z*_*ij*_~*N*(0, 1). This simulates an ICC of *ρ* and a ratio of individual level variance between the non-clustered control arm and the clustered intervention arm of *γ*. If *γ* = 1, there is homoscedasticity between the individual level variance in the control and intervention arms. Full simulation study steps, including the data generation process and modelling, are presented in Fig. 1.

Simulation scenarios are presented in Table 4. We varied: the intervention effect, ICC, cluster size, number of clusters, and ratio of individual variance between the trial arms. If *γ* = 0.25 then individual variance in the control arm is one quarter that in the intervention arm and if *γ* = 4 then individual variance in the control arm is four times that in the intervention arm.

**Table 4:** Simulation input scenario values (total 1440 scenarios)

Variable	Notation	Values
Number of clusters	*c*	3, 6, 12, 24
Cluster size	*m*	5, 10, 20, 30
Intervention effect	*θ*	0, 0.2, 0.5
ICC	*ρ*	0, 0.01, 0.05, 0.1, 0.2^a^, 0.3^a^
Ratio of individual variance between control and cluster trial arms	*γ*	0.25^a^, 0.5, 1, 2, 4^a^

^a^Considered extreme values to occur in rare scenarios

**Fig. 2:**
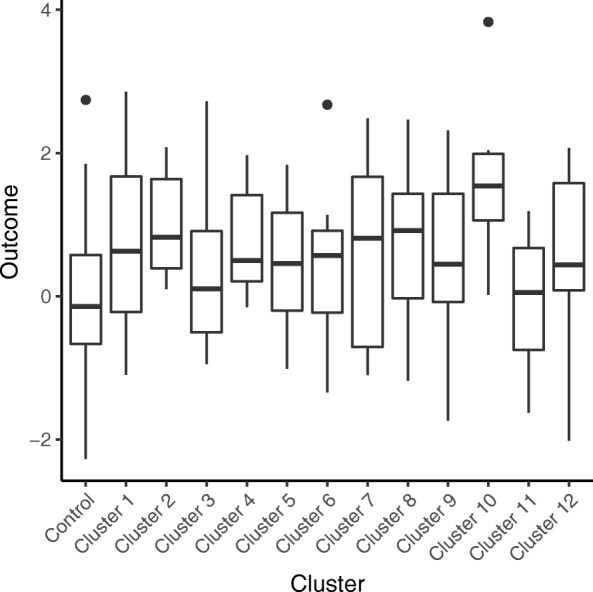
Example of simulated partially nested trial dataset, *ρ* = 0.1, *γ* = 1, *c* = 12, and *m* = 10

Simulation values were chosen based on literature on pnRCTs [7, 9, 15, 17, 18, 26–28], as well as extending these to more extreme cases of *γ* and *ρ* that may occur in rare instances. Reporting of ICCs in iRCTs with clustering is limited at present and it is plausible that ICCs in pnRCTs differ from those of cRCTs. Current evidence suggests ICCs in iRCTs with clustering in either one or both arms are generally small and often less than 0.05 [7–9, 29], hence the choice to include a small ICC *ρ* = 0.01 in the simulations with ICCs of 0.2 or more occurring only in rare instances. We were unaware of specific literature on the evidence of heteroscedasticity, however, from the authors experience of working on trials it was expected *γ* to typically stay within the range of 0.5–2. The number of clusters in the intervention group was 3, 6, 12 or 24. These figures reflect the small numbers of clusters recruited in many pnRCTs and, coupled with the cluster sizes of 5, 10, 20 or 30, they allowed alternative combinations of cluster size and number of clusters to be investigated for a given total trial sample size. Figure 2 provides a graphical example of the simulated partially nested trial data.

## Methods

Each simulated dataset was analysed using the models described in Table 2.

### Estimand

Our estimands of interest are the REML estimate of the intervention effect *θ* and the model estimate of the ICC *ρ*.

### Performance measures

We used the following performance measures: bias, mean square error (MSE), and coverage of 95% confidence intervals for $\widehat{\theta}$, Type I error rate and power (calculated as the proportion of simulated results with a statistically significant intervention effect at the 5% level when the null or alternative hypothesis were true, for Type I error and power respectively) and where applicable, model estimated ICC. See Additional file 2 for more detail on performance measures. For each of the 1440 scenarios 1000 datasets were generated; a 5% significance level and 95% confidence interval based on 1000 simulations has a Monte Carlo error of 0.7%.

## Results

Model convergence was generally satisfactory for all models with models converging 95–100% of the time across the different scenarios.

### Imposed clustering in the control arm

Methods for imposing clustering in the control arm, presented in Table 3, had a negligible impact on the performance measures of the partially nested mixed-effects models (models 3 and 4). Under the simulation scenarios, the differences in the *p*-value, confidence intervals and estimated ICC between the methods were only present at four decimal places. Model fitting was considerably faster (around four to five times faster) using either one large cluster or the pseudo clusters compared to the singleton clusters, however, this will likely be immaterial when fitting only a small number of models.

Methods for imposing clustering in the control arm had a large impact on the performance measures of the fully clustered mixed-effects models (models 2.1, 2.2, and 2.3). Specific results for each performance measure are presented in the following sections.

Results are reported only for the partially nested mixed-effects models (models 3 and 4) with the non-clustered controls classified as one large cluster, as other methods were comparable. All three methods for classifying the non-clustered control arms for the fully clustered mixed-effects model (models 2.1, 2.2, and 2.3) are reported.

### Bias

The bias of the intervention effect estimate was not affected by the analysis model used, individual variances (*γ*) or the ICC (*ρ*). The maximum absolute bias of the intervention effect was |0.057|, |0.043|, and |0.053| for *θ* = 0, 0.2 and 0.5, respectively.

### Mean square error

The models produced unbiased estimators with no difference in the observed MSE between the different models. The MSE of the intervention effect estimate had a mean of 0.051 (standard deviation (SD) 0.056) and maximum of 0.346.

### Type I error

**Fig. 3:**
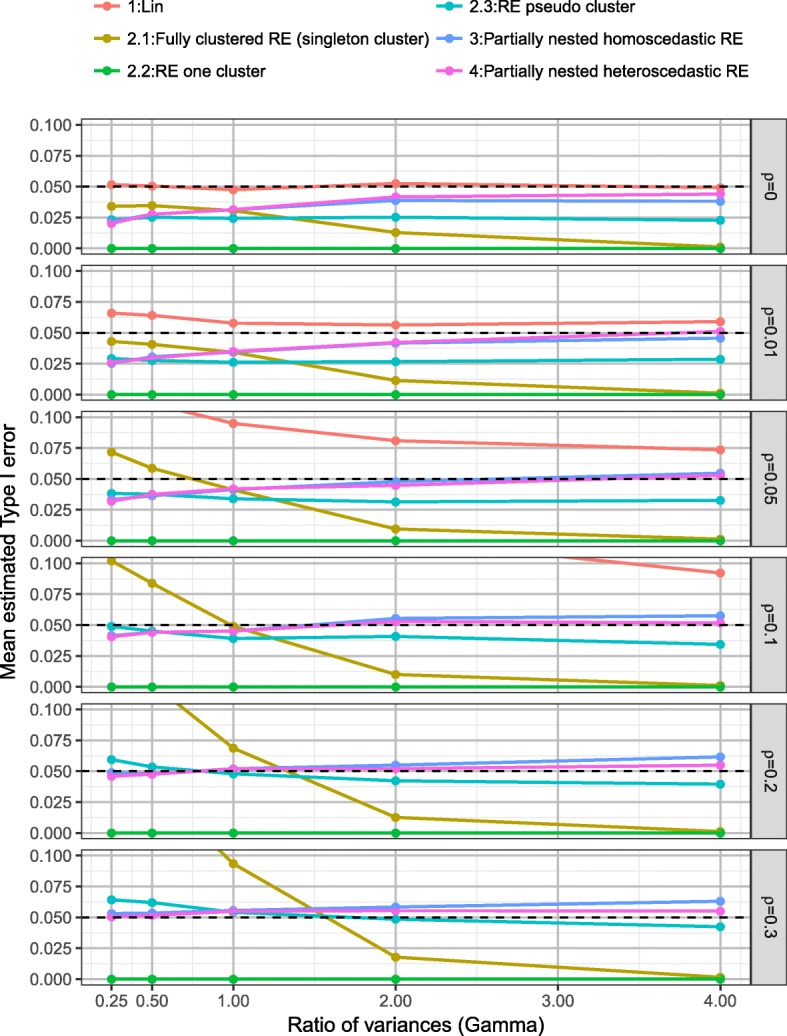
Mean Type I error rate by *γ* and *ρ* over all scenarios, for each model

Plots of the mean Type I error rates split by model, the ratio of individual variances (*γ*) and the ICC (*ρ*) are presented in Fig. 3. As would be expected the linear regression model which ignores clustering had inflated Type I error rates, with Type I error rate affected by ICC (*ρ*), the ratio of individual variances (*γ*)*,* number of clusters (*c*), and cluster size (*m*). Although the inflation was minimal when ICC *ρ* = 0.01, the mean Type I error was 0.061 (SD 0.010). When cluster size *m* ≤ 10 and ICC *ρ* = 0.01 the mean Type I error rate was 0.056 (SD 0.007).

Model 2, the fully clustered models with imposed clustering in the control arm resulted in biased Type I error rates. Imposing clustering as singleton clusters (model 2.1) led to Type I error rates which were largely affected by the ratio of individual variances (*γ*) and ICC (*ρ*). Imposing one large cluster in the control arm (model 2.2) resulted in Type I error rates of zero (due to the Satterthwaite degrees of freedom correction resulting in large degrees of freedom when imposing one large cluster in the control arm). Imposing pseudo clusters in the control arm of the same size as the intervention arm (model 2.3) provided relatively good control of Type I error rates, mean Type I error of 0.039 (SD 0.018), but was affected slightly by both the ratio of individual variances (*γ*) and ICC (*ρ*).

Both the homoscedastic and heteroscedastic partially nested models (models 3 and 4) provided good control of Type I error rates (model 3: mean Type I error 0.045 (SD 0.016) and model 4: mean Type I error 0.044 (SD 0.014)) with little difference present between the two models.

**Fig. 4:**
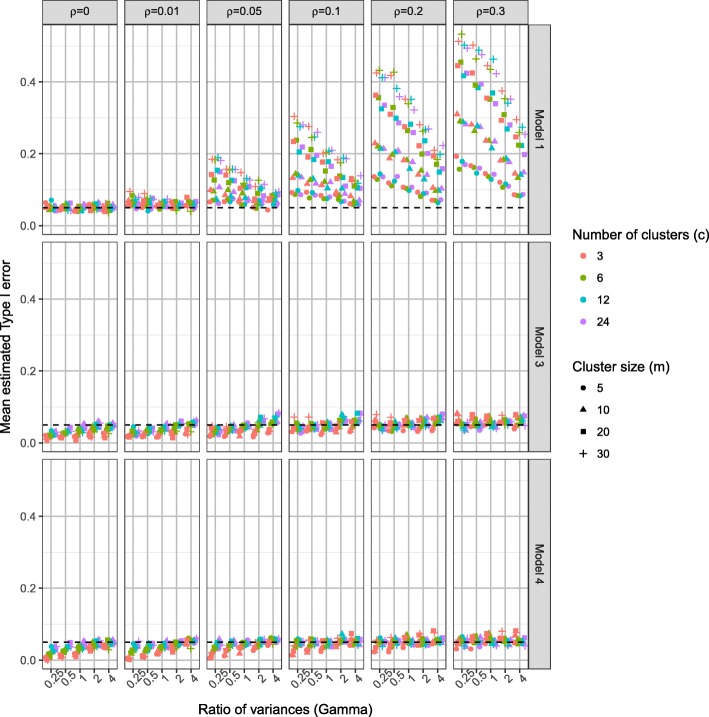
Type I error rate of models 1, 3 and 4, by *ρ*, *γ*, *c*, and *m*

For more detailed comparison Fig. 4 presents the Type I error rates for the linear regression model (model 1), the homoscedastic (model 3) and the heteroscedastic (model 4) partially nested models by ICC (*ρ*), the ratio of individual variances (*γ*)*,* number of clusters (*c*), and cluster size (*m*). Higher ICC values resulted in higher Type I error rates in each model. The impact of ignoring clustering (model 1) depends on both ICC (*ρ*)*,* cluster size (*m*), and number of clusters (*c*). Larger number of clusters (*c*) resulted in better control of Type I error rates for the partially nested models. When ICC *ρ* = 0, the Type I error rates of the partially nested models (models 3 and 4) were reduced from the nominal level. This is due to the cluster variance components being estimated when they are not actually present in the data. When the ICC was small (*ρ* ≤ 0.05) and the individual variance in the control arm smaller than that in the intervention arm (*γ* < 1), the Type I error rates of partially nested models were reduced from the nominal 5% level. When ICC was large (*ρ* = 0.3) the partially nested models generally resulted in inflated Type I error rates. As ICC increased Type I error rates increased, with the partially nested models 3 and 4 only reaching above the nominal Type I error rate of 5% on average when ICC *ρ* ≥ 0.2.

The Satterthwaite correction used in Stata mixed (dfmethod(sat)) did not fully correct the Type I error rates to the nominal 5% level, even with the use of the heteroscedastic model 4. The heteroscedastic model 4 did have slightly improved control of Type I error rates than the homoscedastic model 3.

### Coverage

**Fig. 5:**
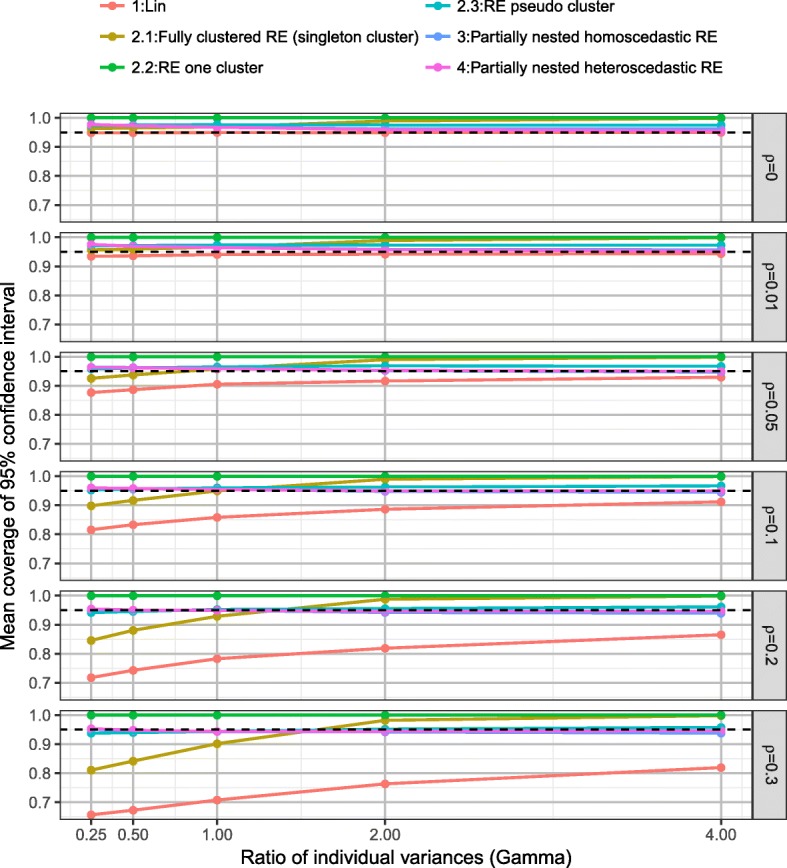
Mean coverage of 95% confidence interval, by *ρ* and *γ* over all scenarios

Plots of the mean coverage of the 95% confidence intervals of the intervention effect estimate split by model, ICC (*ρ*) and the ratio of individual variances (*γ*) are presented in Fig. 5 under the alterative hypothesis. The linear regression model (model 1) resulted in under coverage when ICC was small (*ρ* ≤ 0.05) and the coverage rates decrease as ICC (*ρ*) increases. The fully clustered models with imposed clustering in the control arm resulted in both over and under coverage dependent on the direction of the variance ratio and the method of imposed clustering. Imposing clustering as singleton clusters (model 2.1) resulted in coverage rates largely affected by ratio of individual variances (*γ*). Imposing one large cluster in the control arm (model 2.2) resulted in over coverage, due to the reduced Type I error rates of zero caused by the Satterthwaite degrees of freedom correction. Imposing pseudo clusters in the control arm (model 2.3) provided mean coverage rates of 0.961 (SD 0.018).

Both the homoscedastic and heteroscedastic partially nested models (models 3 and 4) provided good control of coverage rates of 95% confidence intervals (model 3: mean coverage rate 0.956 (SD 0.014) and model 4: mean coverage rate 0.956 (SD 0.014)) with little difference between the two models. In the simulations over coverage of the 95% confidence intervals for the heteroscedastic model 4 occurred when ICC *ρ* ≤ 0.05, except when the ratio of individual variances *γ* = 4. Hence, the results were generally conservative when ICC was small. Under coverage of the 95% confidence intervals for the heteroscedastic model 4 only occurred for large ICC (*ρ*) and ratio of individual variances (*γ*).

### Power

**Fig. 6:**
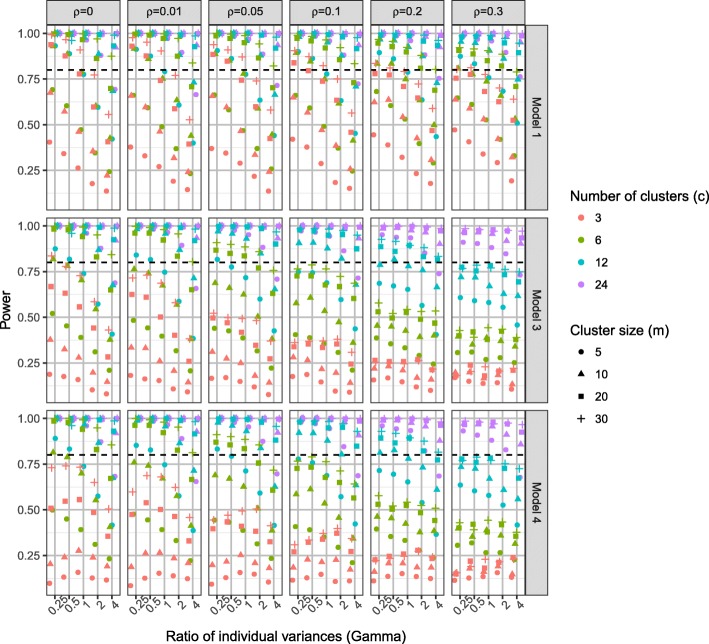
Power when *θ* = 0.5, by *ρ*, *γ*, *c*, and *m*

Increasing the number of clusters as opposed to increasing the cluster size had a bigger impact on power with a fixed total sample size. Fig. 6 shows the power of the linear regression model (model 1), the homoscedastic (model 3) and the heteroscedastic (model 4) partially nested models when intervention effect *θ* = 0.5 by ICC (*ρ*), the ratio of individual variances (*γ*)*,* number of clusters (*c*), and cluster size (*m*) (see Additional file 2 for when *θ* = 0.2). Under the simulation scenarios conducted, 12 or more clusters and cluster sizes of ten or more were generally needed for a power greater than 80%. Using three or six clusters rarely gave power over 80%, only for ICC *ρ* ≤ 0.05 and relatively large cluster sizes *m* ≥ 20, did power go over 80%.

For ICC *ρ* ≤ 0.05, which is commonly assumed when planning complex intervention trials in healthcare, power of 80% was generally achieved with: 24 clusters of any size, 12 clusters of size ten or more, and six clusters of size 20 or more (120 in each arm).

**Fig. 7:**
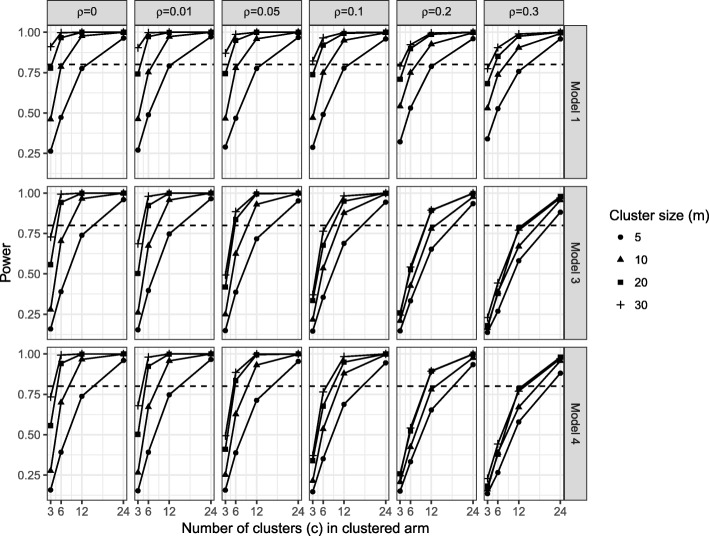
Power with standardised intervention effect of 0.5 (*θ* = 0.5 and *γ* = 1)

Under a ratio of individual variances *γ* = 1 the total residual variance in both trial arms is equal to one, hence, the intervention effect (*θ*) we simulated is the standardised intervention effect. Figure 7 shows the power of models 1, 3 and 4 under homoscedastic individual variances (*γ* = 1). The heteroscedastic model 4 is over-parameterised in the case of the ratio of individual variances *γ* = 1, however, it did not result in a substantially lower power than the homoscedastic model.

Table 5 presents the power of model 4 and model 1 under ICC *ρ* = 0, model 4 is over-parametrised here. There is a loss in mean statistical power which ranged between 1.7 to 6.3%.

**Table 5:** Mean and SD of power of model 4 versus model 1 under *ρ* = 0 over all scenarios

Intervention effect (θ)	Model	Power
		Mean (SD)
0	1	0.050 (0.007)
	4	0.033 (0.014)
0.2	1	0.388 (0.276)
	4	0.327 (0.286)
0.5	1	0.803 (0.254)
	4	0.740 (0.298)

### ICC

**Fig. 8:**
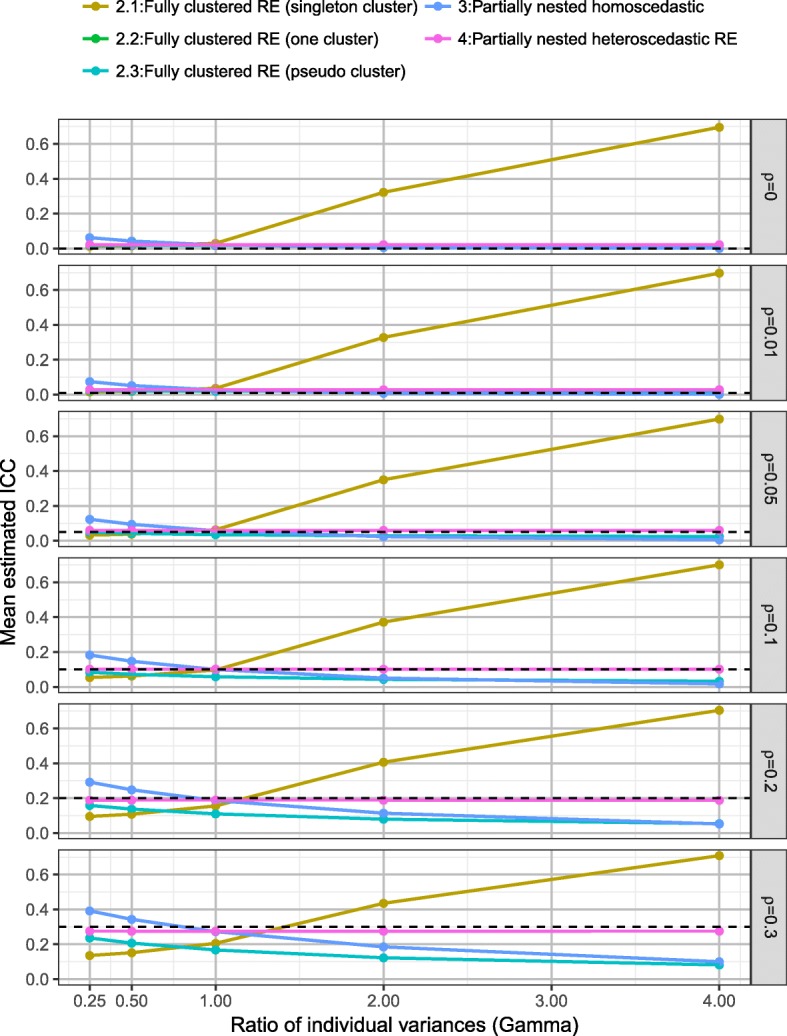
Mean estimated ICC by *γ* and *ρ* over all scenarios, for each model

Figure 8 presents the mean estimated ICC across the fully clustered and partially nested mixed effect models, by the ratio of individual variances (*γ*) and ICC (*ρ*). ICC estimation was consistent under the heteroscedastic partially nested model (model 4). The homoscedastic partially nested model (model 3) resulted in biased ICC, with the direction of bias dependent upon the ratio of individual variances (*γ*).

**Fig. 9:**
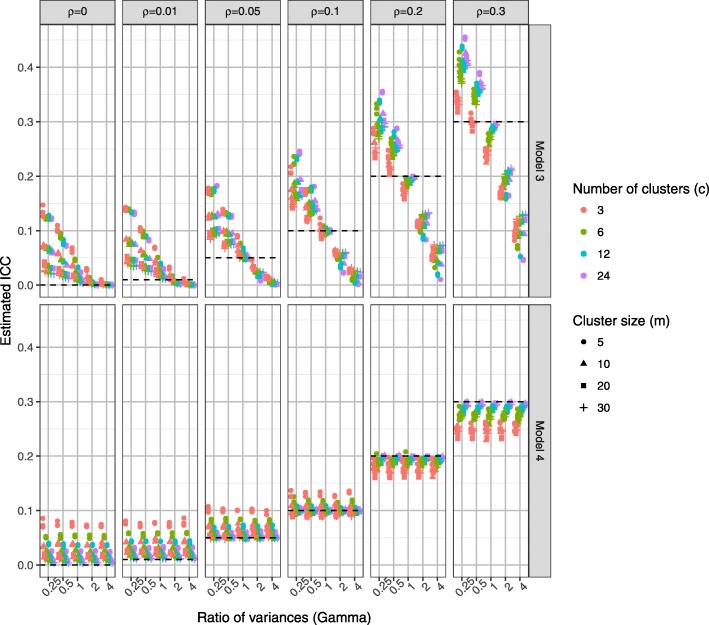
ICC estimation of heteroscedastic partially nested model, by *γ*, *ρ*, *m* and *c*

Figure 9 presents the ICC for the homoscedastic (model 3) and heteroscedastic (model 4) partially nested models by the ratio of individual variances (*γ*), ICC (*ρ*), number of clusters (*c*), and cluster size (*m*). The ICC estimation from the homoscedastic model was highly affected by *γ*. The ICC estimation from the heteroscedastic model was not affected by *γ*. Using the heteroscedastic model, there was a slight positive bias in the ICC estimation when ICC *ρ* ≤ 0.05, and when ICC *ρ* ≥ 0.2 there was slight negative bias in the ICC estimation. For example, when ICC *ρ* = 0.0 the mean ICC estimation was 0.028 (SD 0.018), and when ICC *ρ* = 0.05 the mean estimation was 0.060 (SD 0.014). As expected ICC estimation improved as sample size increased. The ICC estimation was only consistent across all values of ICC (*ρ*) when there were 24 clusters, regardless of cluster size. For an accurate estimate of ICC when true ICC *ρ* = 0.05, under the simulation scenarios we required cluster sizes (*m*) of 20 or 30 or at least six clusters of size ten or 24 clusters of size five.

### Summary of results

Simulation results are summarised in Table 6 presenting the performance of the simple linear regression model (model 1), homoscedastic partially nested mixed effects model (model 3) and heteroscedastic partially nested mixed effects model (model 4) under different design scenarios. Results from the fully clustered mixed-effects models (model 2) are excluded from Table 6 as we do not recommend these in any scenario regardless of the method used to impose clustering in the control arm. None of the fully clustered mixed-effects models provided full control of the Type I error rates and the partially nested mixed effects models always outperformed them.

**Table 6:**
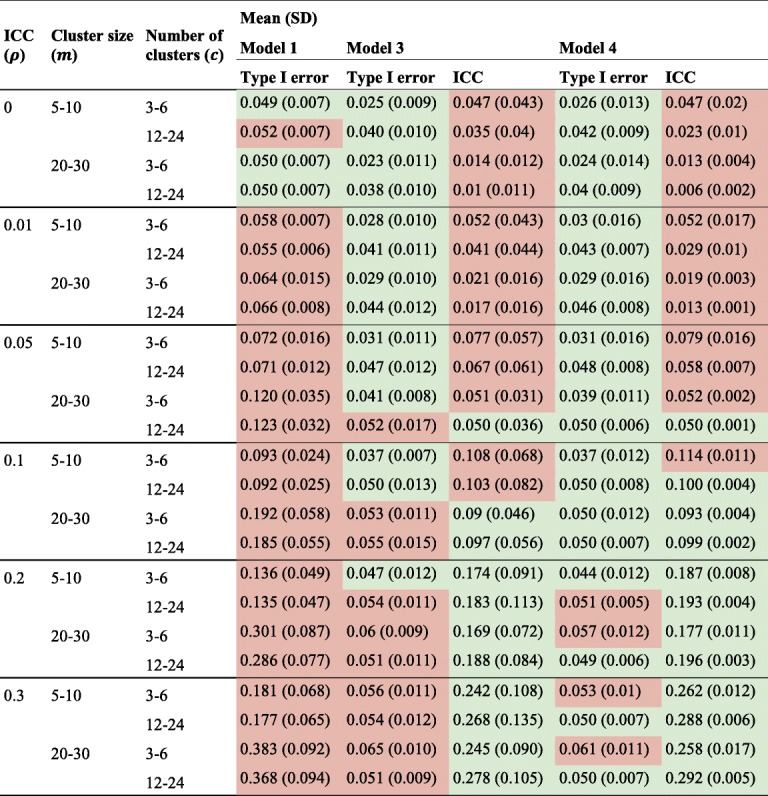
Summary of simulation results by different models split by *ρ*, *m*, and *c* averaged over all *γ*

^*^Model 1: simple linear regression; Model 3: homoscedastic partially nested mixed effects model; Model 4: heteroscedastic partially nested mixed effects model. Green highlighted ≤ than expected, red highlighted > than expected

## Discussion

In this study, we have investigated six modelling strategies for the analysis of pnRCTs with a continuous outcome. Our simulation study showed that when analysing pnRCTs the use of the heteroscedastic partially nested mixed-effects model for normally distributed outcome data (using Satterthwaite degrees of freedom) in general provides: unbiased effect estimates; maintains relatively good control of Type I error rates; and did not noticeably cause a reduction in power even with homoscedastic individual variances across arms. The heteroscedastic partially nested model takes account of the between-cluster variance (if present) and therefore provides valid inferences for the intervention effect. Additional file 2 presents model-fitting code for R, Stata and SAS. When using the partially nested mixed-effects model, the method of classifying the non-clustered controls had a negligible impact on statistical inference under the simulation scenarios, agreeing with findings from analysis of four example pnRCTs by Flight et al. [9].

Our findings were broadly similar to those of Baldwin et al. [15]. However, they did not assess the method of classifying the non-clustered controls or performance of models under small ICC (*ρ* = 0.01, lowest value used in our study) which commonly occur in pnRCTs [7–9, 26, 29]. Unlike findings from Baldwin et al. [15], the Satterthwaite degrees of freedom correction did not fully control the Type I error rate in our simulations. The largest discrepancy from the nominal level occurred when the ICC was small, ratio of individual variances <1, and under small sample sizes.

We have illustrated that using a naïve linear regression model, which ignores clustering in pnRCTs, gave inflated Type I error rates and resulted in under coverage of confidence intervals when clustering of outcomes was present. When ICC 0.01 ≤ *ρ* ≤ 0.05, which we believe is common in pnRCTs [9], ignoring clustering led to largely inflated Type I error rates using the linear regression model. A low ICC may still have a large impact, particularly when cluster sizes are large.

When ICC was small and/or with very few clusters and small cluster sizes using the partially nested mixed-effects models 3 and 4 resulted in underestimated Type I error rates. These models correctly reflect the design of the trials; however, they can result in conservativism regarding the precision of estimates due to the bias in estimating the variance estimates when we have a small number of clusters. Consequently, using the partially nested mixed effects models with small ICC may make it difficult to detect differences between the trial arms when present.

Sanders [17] recommend evaluating whether ICC is significantly different from zero prior to selecting an analysis method. We caution such significance testing for ICC, and similarly testing for heteroscedasticity [7]. These tests will generally lack power in a pnRCT and it is not the statistical significance of the ICC that matters but impact of the magnitude on inference. In general, we recommend the use of the partially nested models when analysing pnRCT trials, particularly if conservatism and an ICC estimate are desired. However, the choice of model and the requirement or not for conservatism needs to be considered in the context of the specific trial setting.

Similar to cRCTs [1], in a pnRCT increasing the number of clusters rather than increasing the cluster size has a greater increase in power for a fixed total sample size. Our simulation results showed that this will also provide a more accurate estimation of the ICC. When the number of clusters is small, for example, three clusters in the intervention arm, the ICC estimation will likely be upwardly biased. With six clusters in the intervention arm, the ICC estimate was relatively unbiased once the true ICC ≥0.1. The ICC estimation became consistent regardless of cluster size or true ICC only once there were 24 clusters in the simulation scenarios. This reflects findings from previous research that to reliably estimate the size of clustering effects a large number of clusters are required [30].

This study investigated the case of analysing partially nested trials under complete compliance. Non-compliance in the clustered arm of a pnRCT may occur when some participants randomised to a particular treatment group or care provider do not attend any sessions or receive treatment as part of different treatment group or care provider intended at randomisation. Consequently, non-complier outcomes may be assumed independent if they do not receive the clustered intervention. Schweig and Pane [16] describe and compare models for pnRCTs with non-compliance using a simulation study. They argue that an unbiased intention-to-treat (ITT) estimate under non-compliance on a pnRCT may be obtained using a Complier Average Causal Effects (CACE) model. This method involves estimating the treatment effect for compliers and scaling this CACE effect estimate by the proportion of compliers to provide an ITT effect estimate. The issues posed by non-compliance warrant further investigation, considering a broader range of scenarios and investigating the degrees of freedom corrections for valid statistical inferences.

The design and analysis of trials with clustering in one arm needs to take account of heterogeneity and ICC to have a sufficiently powered sample size and accurate intervention effect. We strongly recommend the reporting of ICCs in trials results papers. The framework developed for cRCTs is also broadly applicable in iRCTs with clustering, identifying three dimensions to consider when reporting an ICC: a description of the dataset (including characteristics of the outcome and the intervention); how the ICC was calculated; and the precision of the ICC [31]. This has the potential to improve the assumptions about ICCs in iRCTs, adhere to CONSORT reporting guidelines for RCTs of nonpharmacological interventions [32], and raise awareness of the need to account for clustering in both the sample size and analysis in iRCTs with clustering.

A wide variety of terminology are used in iRCTs with clustering in one arm, including partially nested, partially clustered, multi-level, and individually randomized group intervention. A more consistent use of terminology would reduce confusion, improve reporting and make finding relevant ICCs from previous trials easier. We suggest the terminology partially nested randomised trial (pnRCT) to describe an iRCT with clustering in one arm.

### Limitations

All the mixed-effects models assume that the cluster level means follow a Normal distribution. This may not be a valid assumption, for example, when we have a small number of clusters.

In the simulations, we have used fixed cluster sizes. In practice, cluster size may vary, causing a loss in efficiency when estimating the intervention effect. A simulation study by Candel and Van Breukelen [10] found the efficiency loss in the intervention effect estimate was rarely more than 10%, requiring recruitment of 11% more clusters for the intervention arm and 11% more individuals for the control arm. The loss of efficiency in the intercept variance reached to 15%, requiring 19% more clusters in the clustered arm, and no additional recruitment in the control arm. Additionally, it has been shown in cluster trials if the coefficient of variation in cluster size is small, less than 0.23, then the correction on sample size is negligible [33]. It should be noted that cluster sizes are likely to be more similar in group administered interventions compared to trials which impose clustering by being treated by the same care provider [7].

Throughout the simulations we assumed there was no effect of clustering in the control arm, this may not strictly be true in practice. In healthcare intervention trials, a commonly used control intervention is ‘care as usual’. This type of control may induce some form of low-level clustering, for instance, treatment by a healthcare practitioner. If the same practitioner treats numerous individuals, we can assume, in the same sense as we have done for the intervention arm that these individuals are clustered and include this in the modelling procedure. However, this information is often not available in trial data and is not unique to pnRCTs.

Partially nested trials pose a number of challenges, in particular, the issue of internal validity [6]. The grouping of individuals as part of the delivery of a treatment may affect the outcome. However, taking a pragmatic viewpoint, we consider the grouping as part of the treatment as a whole if this is reflective of treatment delivery in real-world practice. In addition, if the ungrouped controls are the true comparison in real life a pnRCT design will provide external validity.

## Conclusion

Partially nested RCTs are increasingly used in complex intervention research. Ignoring clustering can lead to inflations of the Type I error rates, even for small ICCs. When analysing a pnRCT with continuous outcomes we recommend the use of a heteroscedastic partially nested mixed-effects model with corrected degrees of freedoms such as using the Satterhwaite method, for outcomes similar to those generated under the scenarios of our simulations study. The method used for classifying the non-clustered controls had a negligible impact on the results using the partially nested mixed-effects model. The model is easy to implement in standard statistical software and does not cause a notable reduction in power under homoscedastic variances. With few clusters, small cluster sizes and small ICC, the partially nested model underestimated Type I error rates and gave largely inflated ICC estimates, hence, for such designs there is no optimal model and we need to be cautious in model interpretation. Finally, to aid the design and prior selection of an appropriate analysis plan for pnRCTs, we strongly recommend the reporting of estimated ICC when publishing trials results.

## Additional files

Additional file 1: Example Stata code used to run the simulations described in the manuscript text. (DOCX 16 kb)

Additional file 2: Additional details including: model fitting code for Stata, R, and SAS for the homoscedastic and heteroscedastic partially nested models; performance measures; and results tables. (DOCX 189 kb)

## Abbreviations

cRCT: Cluster randomised controlled trials
ICC: Intracluster correlation coefficient
iRCT: Individually randomised controlled trials
pnRCT: Partially nested randomised controlled trials
