# Supplementary material for: Appropriate statistical methods for analysing partially nested randomised controlled trials with continuous outcomes: a simulation study
Source: BMC Med Res Methodol. 2018 Oct 11;18:105. doi: 10.1186/s12874-018-0559-x (PMC6186141; doi:10.1186/s12874-018-0559-x)
Supplement: Supplementary file 1 — Example Stata code used to run the simulations described in the manuscript text. (DOCX 16 kb) [file 12874_2018_559_MOESM1_ESM.docx]

# Supplementary material

### **Model fit code for Stata, R, and SAS**

It is still common for clustering in pnRCTs to be ignored in the design and analysis stages. To encourage the use of analysis methods which take account of clustering eTable1 presents commands to implement the homoscedastic and the heteroscedastic partially nested models in three commonly used statistical packages, Stata, R, and SAS, along with the degrees of freedom correction options (where available). The homoscedastic model is included for clarity and where there is a strong priori belief of homoscedasticity it may be more suitable to use. Both the homoscedastic and heteroscedastic models can be fitted in Stata and SAS with the option of the Satterthwaite degrees of freedom correction. Both models can be fitted in R, however, at the time of submission of this manuscript we are unaware of a method that allows the fitting of the heteroscedastic partially nested model (model 4) using the Satterthwaite degrees of freedom correction. Instead, it is possible to use bootstrapping to obtain confidence intervals and the likelihood ratio test to obtain p-values for the effect estimate.

**eTable 1:** Stata, R and SAS model fitting commands for the partially nested models

| **Software** | **Homoscedastic partially nested model** | **Heteroscedastic partially nested model** |
| --- | --- | --- |
| Stata | *mixed* command with *dfmethod(sat)* option | mixed command with dfmethod(sat) option and residuals(independent, by(intervention)) |
| Example code | mixed y treat \|\| cluster:treat, nocons reml dfmethod(sat) | mixed y treat \|\| cluster:t, nocons reml residuals(independent, by(treat)) dfmethod(sat) |
| R* | *lmer* function from *lme4* package, and package *lmerTest* for Satterthwaite df | *lme* function from *nlme* package with *weights=varIdent(form=~1\|treat)* option. No option for Sattertwaite df. |
| Example code | lmer(y ~ x + (0 + treat\|cluster)) | lme(fixed=y ~ treat, random=~(0+treat)\|cluster,  weights=varIdent(form=~1\|treat),  method ="REML") |
| SAS | *proc mixed* command with the option *ddfm=sat* | proc mixed command with the option ddfm=sat and repeated / group = treat; |
| Example code | proc mixed covtest;    class cluster treat;    model y = treat / solution ddfm=sat;    random intercept treat / subject = cluster;  run; | proc mixed covtest;    class cluster treat;    model y = treat / solution ddfm=sat;    random intercept treat / subject = cluster;    repeated / group = treat;  run; |
| y = outcome, treat = intervention arm indicator, cluster = intervention cluster indicator.  *At present cannot fit the heteroscedastic partially nested model with degrees of freedom correction in R. The function *lmer* in R does not allow for different variances for each level of a grouping factor. | | |

### **Performance measures used to assess results**

The simulation results were assessed in terms the following performance measures:

- Bias of the effect estimate: calculated as the difference between the average estimate of the effect and the true effect using $\text{Bias}=\text{E}\left( \hat{\theta} \right)-\theta$.
- Mean squared error: provides a measure of accuracy which incorporates both bias and variability and calculated using $MSE=\text{E}\left[ \left( \hat{\theta}-\theta\right)^{2} \right]$
- Type I error rate: proportion of simulations in which the p-value <0.05 when the null hypothesis is true, true intervention effect$\theta=0$.
- Coverage of the 95% confidence intervals: proportion of simulations that the obtained 95% confidence interval contains the true specific intervention effect$\theta$.
- Power: proportion of simulations in which the p-value <0.05 when the alternative hypothesis is true, true intervention effect$\theta\neq0$.
- Intracluster correlation coefficient: calculated using $\hat{\rho}={\hat{\sigma_{u}^{2}}}/{\hat{\sigma_{\epsilon}^{2}}+\hat{\sigma_{u}^{2}}}$, where $\hat{\sigma_{\epsilon}^{2}}$ and $\hat{\sigma_{u}^{2}}$ are within- and between-cluster variance estimates for the clustered intervention arm.

## Supplementary figures and tables

**eFigure 1**: Bias of effect estimate by $\theta$ and model


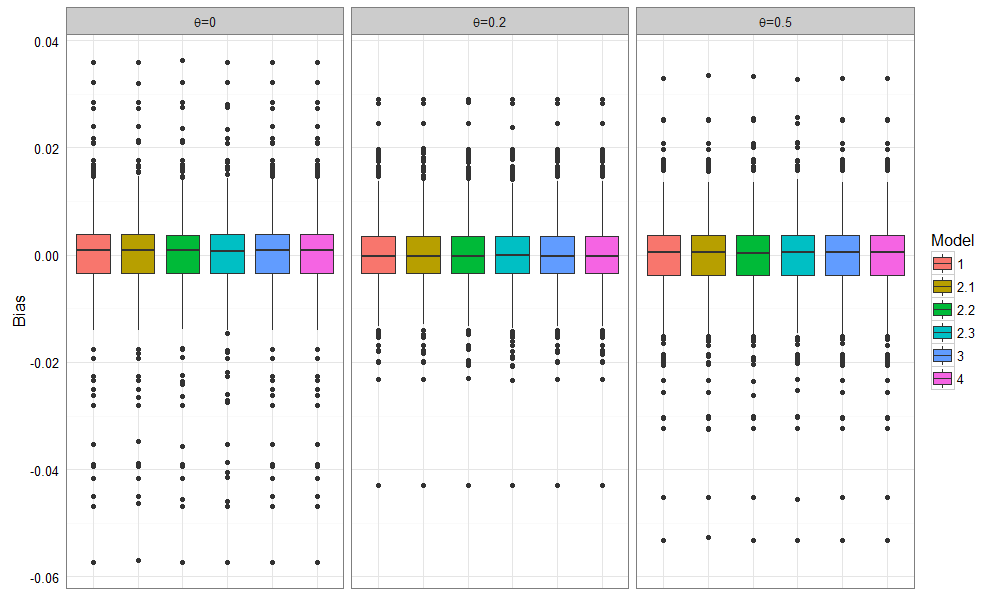


Models produced unbiased estimators and there was no difference in the relationship observed for MSE between the different. Therefore, we only present model 4 the heteroscedastic partially clustered model in eFigure 2. The high MSEs were observed for small sample sizes (small clusters and small numbers of clusters).

MSE increased as $\rho$ increased and was also affected by $\gamma$. Higher $\rho$ values reduced power due to the lack of independence of the data, resulting in reduced accuracy shown by the MSE. We simulated data to induce a fixed $\rho$ and not a fixed variance, therefore, as γ increases the total variance in the control arm increases as we move from $\gamma=0.25$ to $\gamma=4$. The increase in MSE illustrated in Figure 1 can be explained by the increase in γ increasing the total variance in the control arm.

**eFigure 2:** Mean Square error of intervention effect estimate, split by $\gamma$ and $\rho$


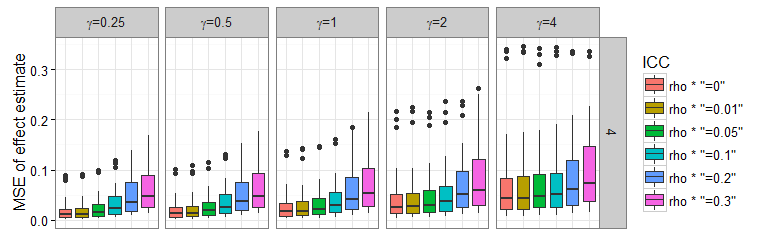


**eFigure 3:** Power of model 1, 3 and 4 when $\theta=0.2$, by $\gamma, \rho, m$ and $c$


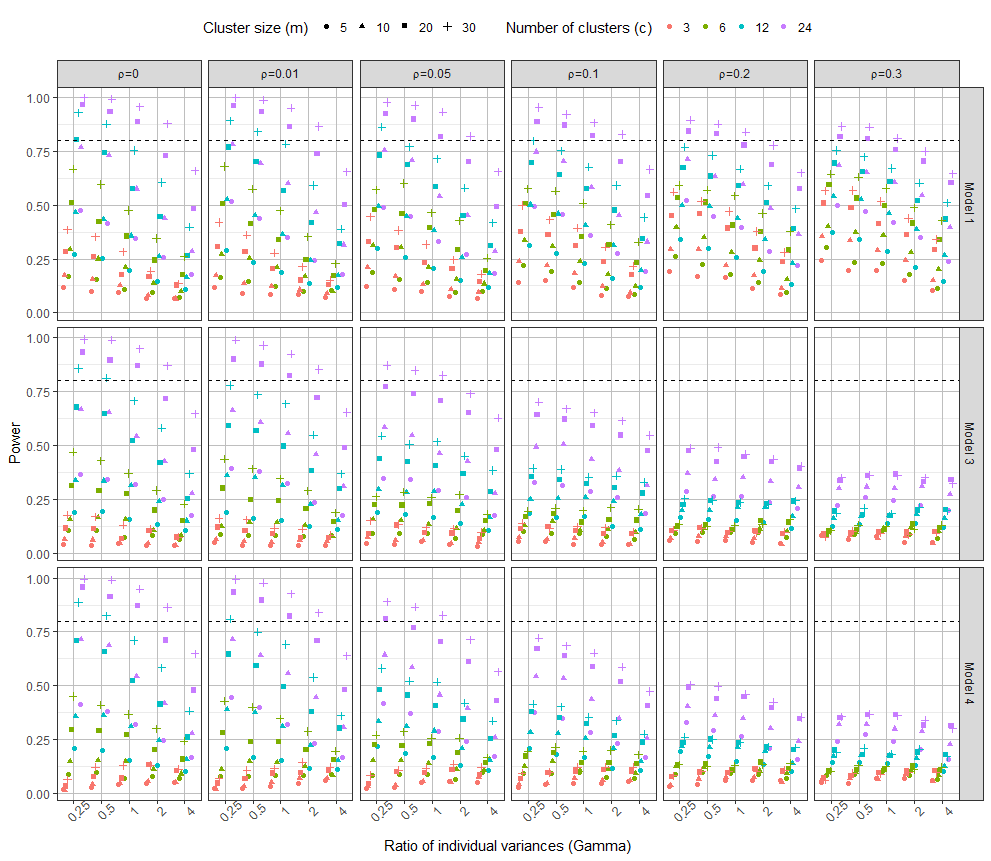


**eFigure 4:** Bias of between- and within-cluster variance estimates from the heteroscedastic partially nested model (model 4) by $\gamma, \rho, m$ and $c$


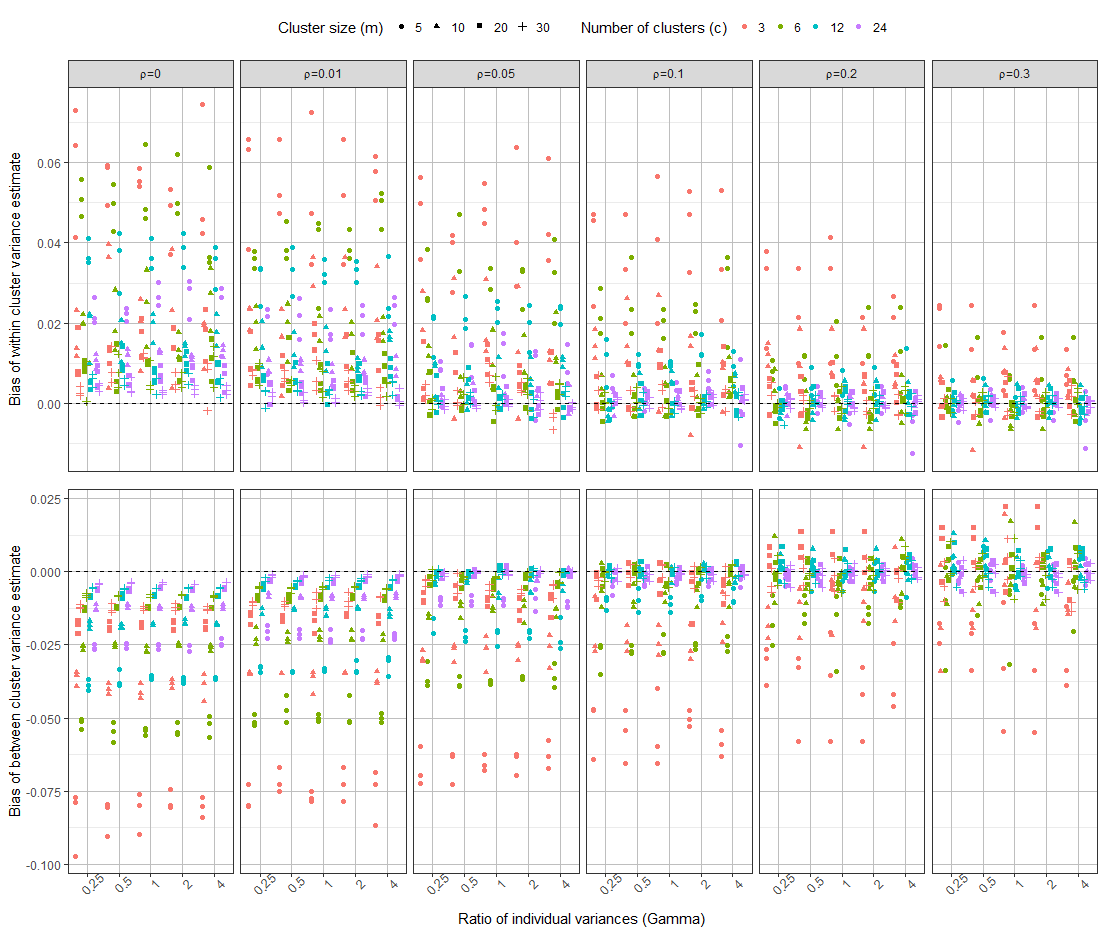


| **eTable 2**: Type 1 error mean and standard deviation by $\gamma$and $\rho$   \|  \|  \| **Model** \| \| \| \| \| \| \| \| \| \| \| \| \| --- \| --- \| --- \| --- \| --- \| --- \| --- \| --- \| --- \| --- \| --- \| --- \| --- \| --- \| \| $\boldsymbol{\gamma}$ \| $\boldsymbol{\rho}$ \| **1** \|  \| **2.1** \|  \| **2.2** \|  \| **2.3** \|  \| **3** \|  \| **4** \|  \| \|  \|  \| **Mean** \| **SD** \| **Mean** \| **SD** \| **Mean** \| **SD** \| **Mean** \| **SD** \| **Mean** \| **SD** \| **Mean** \| **SD** \| \| **0.25** \| **0** \| 0.05 \| 0.01 \| 0.03 \| 0.01 \| 0.00 \| 0.01 \| 0.02 \| 0.01 \| 0.02 \| 0.01 \| 0.02 \| 0.01 \| \| **0.01** \| 0.07 \| 0.01 \| 0.04 \| 0.01 \| 0.00 \| 0.01 \| 0.03 \| 0.01 \| 0.03 \| 0.01 \| 0.03 \| 0.02 \| \| **0.05** \| 0.12 \| 0.05 \| 0.07 \| 0.03 \| 0.00 \| 0.01 \| 0.04 \| 0.01 \| 0.03 \| 0.01 \| 0.03 \| 0.02 \| \| **0.1** \| 0.18 \| 0.08 \| 0.10 \| 0.03 \| 0.00 \| 0.01 \| 0.05 \| 0.01 \| 0.04 \| 0.01 \| 0.04 \| 0.01 \| \| **0.2** \| 0.28 \| 0.11 \| 0.15 \| 0.05 \| 0.00 \| 0.01 \| 0.06 \| 0.01 \| 0.05 \| 0.01 \| 0.05 \| 0.01 \| \| **0.3** \| 0.35 \| 0.13 \| 0.19 \| 0.06 \| 0.00 \| 0.01 \| 0.06 \| 0.01 \| 0.05 \| 0.01 \| 0.05 \| 0.01 \| \| **0.5** \| **0** \| 0.05 \| 0.01 \| 0.03 \| 0.01 \| 0.00 \| 0.01 \| 0.03 \| 0.01 \| 0.03 \| 0.01 \| 0.03 \| 0.01 \| \| **0.01** \| 0.06 \| 0.01 \| 0.04 \| 0.01 \| 0.00 \| 0.01 \| 0.03 \| 0.01 \| 0.03 \| 0.01 \| 0.03 \| 0.01 \| \| **0.05** \| 0.11 \| 0.03 \| 0.06 \| 0.02 \| 0.00 \| 0.01 \| 0.04 \| 0.01 \| 0.04 \| 0.01 \| 0.04 \| 0.01 \| \| **0.1** \| 0.17 \| 0.07 \| 0.08 \| 0.03 \| 0.00 \| 0.01 \| 0.05 \| 0.01 \| 0.04 \| 0.01 \| 0.04 \| 0.01 \| \| **0.2** \| 0.26 \| 0.11 \| 0.12 \| 0.04 \| 0.00 \| 0.01 \| 0.05 \| 0.01 \| 0.05 \| 0.01 \| 0.05 \| 0.01 \| \| **0.3** \| 0.33 \| 0.13 \| 0.16 \| 0.05 \| 0.00 \| 0.02 \| 0.06 \| 0.02 \| 0.05 \| 0.01 \| 0.05 \| 0.01 \| \| **1** \| **0** \| 0.05 \| 0.01 \| 0.03 \| 0.01 \| 0.00 \| 0.01 \| 0.02 \| 0.01 \| 0.03 \| 0.01 \| 0.03 \| 0.01 \| \| **0.01** \| 0.06 \| 0.01 \| 0.03 \| 0.01 \| 0.00 \| 0.01 \| 0.03 \| 0.01 \| 0.03 \| 0.01 \| 0.03 \| 0.01 \| \| **0.05** \| 0.09 \| 0.03 \| 0.04 \| 0.01 \| 0.00 \| 0.01 \| 0.03 \| 0.01 \| 0.04 \| 0.01 \| 0.04 \| 0.01 \| \| **0.1** \| 0.14 \| 0.05 \| 0.05 \| 0.01 \| 0.00 \| 0.01 \| 0.04 \| 0.01 \| 0.05 \| 0.01 \| 0.05 \| 0.01 \| \| **0.2** \| 0.22 \| 0.09 \| 0.07 \| 0.02 \| 0.00 \| 0.01 \| 0.05 \| 0.01 \| 0.05 \| 0.01 \| 0.05 \| 0.01 \| \| **0.3** \| 0.29 \| 0.12 \| 0.09 \| 0.02 \| 0.00 \| 0.01 \| 0.05 \| 0.01 \| 0.06 \| 0.01 \| 0.05 \| 0.01 \| \| **2** \| **0** \| 0.05 \| 0.01 \| 0.01 \| 0.01 \| 0.00 \| 0.01 \| 0.03 \| 0.01 \| 0.04 \| 0.01 \| 0.04 \| 0.01 \| \| **0.01** \| 0.06 \| 0.01 \| 0.01 \| 0.01 \| 0.00 \| 0.01 \| 0.03 \| 0.01 \| 0.04 \| 0.01 \| 0.04 \| 0.01 \| \| **0.05** \| 0.08 \| 0.02 \| 0.01 \| 0.01 \| 0.00 \| 0.02 \| 0.03 \| 0.02 \| 0.05 \| 0.02 \| 0.04 \| 0.01 \| \| **0.1** \| 0.12 \| 0.04 \| 0.01 \| 0.01 \| 0.00 \| 0.02 \| 0.04 \| 0.02 \| 0.06 \| 0.01 \| 0.05 \| 0.01 \| \| **0.2** \| 0.18 \| 0.07 \| 0.01 \| 0.01 \| 0.00 \| 0.01 \| 0.04 \| 0.01 \| 0.05 \| 0.01 \| 0.05 \| 0.01 \| \| **0.3** \| 0.23 \| 0.10 \| 0.02 \| 0.01 \| 0.00 \| 0.01 \| 0.05 \| 0.01 \| 0.06 \| 0.01 \| 0.06 \| 0.01 \| \| **4** \| **0** \| 0.05 \| 0.01 \| 0.00 \| 0.00 \| 0.00 \| 0.01 \| 0.02 \| 0.01 \| 0.04 \| 0.01 \| 0.04 \| 0.01 \| \| **0.01** \| 0.06 \| 0.01 \| 0.00 \| 0.00 \| 0.00 \| 0.02 \| 0.03 \| 0.02 \| 0.05 \| 0.01 \| 0.05 \| 0.01 \| \| **0.05** \| 0.07 \| 0.01 \| 0.00 \| 0.00 \| 0.00 \| 0.02 \| 0.03 \| 0.02 \| 0.05 \| 0.02 \| 0.05 \| 0.01 \| \| **0.1** \| 0.09 \| 0.03 \| 0.00 \| 0.00 \| 0.00 \| 0.02 \| 0.03 \| 0.02 \| 0.06 \| 0.02 \| 0.05 \| 0.01 \| \| **0.2** \| 0.14 \| 0.06 \| 0.00 \| 0.00 \| 0.00 \| 0.01 \| 0.04 \| 0.01 \| 0.06 \| 0.01 \| 0.05 \| 0.01 \| \| **0.3** \| 0.18 \| 0.08 \| 0.00 \| 0.00 \| 0.00 \| 0.01 \| 0.04 \| 0.01 \| 0.06 \| 0.01 \| 0.06 \| 0.01 \|   **eTable 3**: Power mean and standard deviation under alternative hypothesis by$\boldsymbol{\gamma}$ and $\boldsymbol{\rho}$   \|  \|  \| **Model** \| \| \| \| \| \| \| \| \| \| \| \| \| --- \| --- \| --- \| --- \| --- \| --- \| --- \| --- \| --- \| --- \| --- \| --- \| --- \| --- \| \| $\boldsymbol{\gamma}$ \| $\boldsymbol{\rho}$ \| **1** \|  \| **2.1** \|  \| **2.2** \|  \| **2.3** \|  \| **3** \|  \| **4** \|  \| \|  \|  \| **Mean** \| **SD** \| **Mean** \| **SD** \| **Mean** \| **SD** \| **Mean** \| **SD** \| **Mean** \| **SD** \| **Mean** \| **SD** \| \| **0.25** \| **0** \| 0.71 \| 0.31 \| 0.67 \| 0.34 \| 0.06 \| 0.18 \| 0.62 \| 0.37 \| 0.62 \| 0.36 \| 0.60 \| 0.38 \| \| **0.01** \| 0.71 \| 0.31 \| 0.66 \| 0.34 \| 0.06 \| 0.20 \| 0.60 \| 0.36 \| 0.60 \| 0.36 \| 0.58 \| 0.38 \| \| **0.05** \| 0.70 \| 0.30 \| 0.64 \| 0.33 \| 0.03 \| 0.08 \| 0.56 \| 0.35 \| 0.54 \| 0.35 \| 0.54 \| 0.36 \| \| **0.1** \| 0.70 \| 0.28 \| 0.62 \| 0.31 \| 0.01 \| 0.02 \| 0.52 \| 0.34 \| 0.49 \| 0.33 \| 0.48 \| 0.35 \| \| **0.2** \| 0.70 \| 0.25 \| 0.60 \| 0.29 \| 0.00 \| 0.01 \| 0.45 \| 0.32 \| 0.42 \| 0.32 \| 0.42 \| 0.33 \| \| **0.3** \| 0.71 \| 0.23 \| 0.59 \| 0.28 \| 0.00 \| 0.01 \| 0.40 \| 0.30 \| 0.36 \| 0.3 \| 0.36 \| 0.31 \| \| **0.5** \| **0** \| 0.68 \| 0.32 \| 0.63 \| 0.35 \| 0.03 \| 0.11 \| 0.58 \| 0.37 \| 0.6 \| 0.36 \| 0.59 \| 0.37 \| \| **0.01** \| 0.67 \| 0.32 \| 0.62 \| 0.35 \| 0.05 \| 0.16 \| 0.57 \| 0.37 \| 0.58 \| 0.36 \| 0.58 \| 0.37 \| \| **0.05** \| 0.67 \| 0.30 \| 0.60 \| 0.33 \| 0.03 \| 0.08 \| 0.53 \| 0.35 \| 0.53 \| 0.34 \| 0.53 \| 0.35 \| \| **0.1** \| 0.67 \| 0.29 \| 0.58 \| 0.32 \| 0.01 \| 0.02 \| 0.50 \| 0.34 \| 0.48 \| 0.33 \| 0.48 \| 0.34 \| \| **0.2** \| 0.68 \| 0.26 \| 0.56 \| 0.30 \| 0.00 \| 0.00 \| 0.43 \| 0.32 \| 0.41 \| 0.32 \| 0.41 \| 0.32 \| \| **0.3** \| 0.68 \| 0.24 \| 0.55 \| 0.29 \| 0.00 \| 0.00 \| 0.39 \| 0.30 \| 0.36 \| 0.30 \| 0.36 \| 0.30 \| \| **1** \| **0** \| 0.62 \| 0.33 \| 0.55 \| 0.36 \| 0.01 \| 0.02 \| 0.52 \| 0.37 \| 0.56 \| 0.36 \| 0.56 \| 0.36 \| \| **0.01** \| 0.62 \| 0.33 \| 0.55 \| 0.36 \| 0.02 \| 0.07 \| 0.51 \| 0.37 \| 0.55 \| 0.36 \| 0.55 \| 0.36 \| \| **0.05** \| 0.62 \| 0.32 \| 0.51 \| 0.34 \| 0.02 \| 0.08 \| 0.49 \| 0.36 \| 0.51 \| 0.34 \| 0.51 \| 0.34 \| \| **0.1** \| 0.62 \| 0.3 \| 0.48 \| 0.33 \| 0.01 \| 0.02 \| 0.45 \| 0.34 \| 0.46 \| 0.33 \| 0.46 \| 0.33 \| \| **0.2** \| 0.64 \| 0.28 \| 0.46 \| 0.32 \| 0.00 \| 0.00 \| 0.41 \| 0.32 \| 0.40 \| 0.32 \| 0.40 \| 0.32 \| \| **0.3** \| 0.64 \| 0.25 \| 0.46 \| 0.30 \| 0.00 \| 0.00 \| 0.37 \| 0.3 \| 0.36 \| 0.30 \| 0.36 \| 0.30 \| \| **2** \| **0** \| 0.54 \| 0.34 \| 0.22 \| 0.29 \| 0.00 \| 0.00 \| 0.44 \| 0.36 \| 0.50 \| 0.35 \| 0.50 \| 0.35 \| \| **0.01** \| 0.54 \| 0.33 \| 0.22 \| 0.29 \| 0.00 \| 0.00 \| 0.44 \| 0.36 \| 0.49 \| 0.35 \| 0.50 \| 0.34 \| \| **0.05** \| 0.54 \| 0.32 \| 0.22 \| 0.29 \| 0.01 \| 0.04 \| 0.42 \| 0.35 \| 0.47 \| 0.34 \| 0.47 \| 0.33 \| \| **0.1** \| 0.55 \| 0.31 \| 0.21 \| 0.28 \| 0.01 \| 0.02 \| 0.40 \| 0.34 \| 0.44 \| 0.33 \| 0.43 \| 0.32 \| \| **0.2** \| 0.57 \| 0.29 \| 0.22 \| 0.28 \| 0.00 \| 0.00 \| 0.37 \| 0.32 \| 0.39 \| 0.31 \| 0.38 \| 0.30 \| \| **0.3** \| 0.59 \| 0.27 \| 0.25 \| 0.27 \| 0.00 \| 0.00 \| 0.34 \| 0.29 \| 0.35 \| 0.29 \| 0.35 \| 0.28 \| \| **4** \| **0** \| 0.43 \| 0.32 \| 0.02 \| 0.04 \| 0.00 \| 0.00 \| 0.34 \| 0.33 \| 0.40 \| 0.33 \| 0.42 \| 0.32 \| \| **0.01** \| 0.43 \| 0.31 \| 0.02 \| 0.04 \| 0.00 \| 0.00 \| 0.34 \| 0.33 \| 0.40 \| 0.33 \| 0.41 \| 0.32 \| \| **0.05** \| 0.44 \| 0.31 \| 0.02 \| 0.04 \| 0.00 \| 0.00 \| 0.33 \| 0.32 \| 0.40 \| 0.32 \| 0.39 \| 0.31 \| \| **0.1** \| 0.45 \| 0.31 \| 0.02 \| 0.05 \| 0.00 \| 0.01 \| 0.33 \| 0.32 \| 0.39 \| 0.31 \| 0.37 \| 0.30 \| \| **0.2** \| 0.48 \| 0.29 \| 0.04 \| 0.07 \| 0.00 \| 0.00 \| 0.31 \| 0.30 \| 0.36 \| 0.29 \| 0.34 \| 0.28 \| \| **0.3** \| 0.50 \| 0.28 \| 0.05 \| 0.10 \| 0.00 \| 0.00 \| 0.30 \| 0.28 \| 0.33 \| 0.28 \| 0.32 \| 0.27 \| |  |  |  |  |  |  |
| --- | --- | --- | --- | --- | --- | --- | --- | --- | --- | --- | --- | --- | --- | --- | --- | --- | --- | --- | --- | --- | --- | --- | --- | --- | --- | --- | --- | --- | --- | --- | --- | --- | --- | --- | --- | --- | --- | --- | --- | --- | --- | --- | --- | --- | --- | --- | --- | --- | --- | --- | --- | --- | --- | --- | --- | --- | --- | --- | --- | --- | --- | --- | --- | --- | --- | --- | --- | --- | --- | --- | --- | --- | --- | --- | --- | --- | --- | --- | --- | --- | --- | --- | --- | --- | --- | --- | --- | --- | --- | --- | --- | --- | --- | --- | --- | --- | --- | --- | --- | --- | --- | --- | --- | --- | --- | --- | --- | --- | --- | --- | --- | --- | --- | --- | --- | --- | --- | --- | --- | --- | --- | --- | --- | --- | --- | --- | --- | --- | --- | --- | --- | --- | --- | --- | --- | --- | --- | --- | --- | --- | --- | --- | --- | --- | --- | --- | --- | --- | --- | --- | --- | --- | --- | --- | --- | --- | --- | --- | --- | --- | --- | --- | --- | --- | --- | --- | --- | --- | --- | --- | --- | --- | --- | --- | --- | --- | --- | --- | --- | --- | --- | --- | --- | --- | --- | --- | --- | --- | --- | --- | --- | --- | --- | --- | --- | --- | --- | --- | --- | --- | --- | --- | --- | --- | --- | --- | --- | --- | --- | --- | --- | --- | --- | --- | --- | --- | --- | --- | --- | --- | --- | --- | --- | --- | --- | --- | --- | --- | --- | --- | --- | --- | --- | --- | --- | --- | --- | --- | --- | --- | --- | --- | --- | --- | --- | --- | --- | --- | --- | --- | --- | --- | --- | --- | --- | --- | --- | --- | --- | --- | --- | --- | --- | --- | --- | --- | --- | --- | --- | --- | --- | --- | --- | --- | --- | --- | --- | --- | --- | --- | --- | --- | --- | --- | --- | --- | --- | --- | --- | --- | --- | --- | --- | --- | --- | --- | --- | --- | --- | --- | --- | --- | --- | --- | --- | --- | --- | --- | --- | --- | --- | --- | --- | --- | --- | --- | --- | --- | --- | --- | --- | --- | --- | --- | --- | --- | --- | --- | --- | --- | --- | --- | --- | --- | --- | --- | --- | --- | --- | --- | --- | --- | --- | --- | --- | --- | --- | --- | --- | --- | --- | --- | --- | --- | --- | --- | --- | --- | --- | --- | --- | --- | --- | --- | --- | --- | --- | --- | --- | --- | --- | --- | --- | --- | --- | --- | --- | --- | --- | --- | --- | --- | --- | --- | --- | --- | --- | --- | --- | --- | --- | --- | --- | --- | --- | --- | --- | --- | --- | --- | --- | --- | --- | --- | --- | --- | --- | --- | --- | --- | --- | --- | --- | --- | --- | --- | --- | --- | --- | --- | --- | --- | --- | --- | --- | --- | --- | --- | --- | --- | --- | --- | --- | --- | --- | --- | --- | --- | --- | --- | --- | --- | --- | --- | --- | --- | --- | --- | --- | --- | --- | --- | --- | --- | --- | --- | --- | --- | --- | --- | --- | --- | --- | --- | --- | --- | --- | --- | --- | --- | --- | --- | --- | --- | --- | --- | --- | --- | --- | --- | --- | --- | --- | --- | --- | --- | --- | --- | --- | --- | --- | --- | --- | --- | --- | --- | --- | --- | --- | --- | --- | --- | --- | --- | --- | --- | --- | --- | --- | --- | --- | --- | --- | --- | --- | --- | --- | --- | --- | --- | --- | --- | --- | --- | --- | --- | --- | --- | --- | --- | --- | --- | --- | --- | --- | --- | --- | --- | --- | --- | --- | --- | --- | --- | --- | --- | --- | --- | --- | --- | --- | --- | --- | --- | --- | --- | --- | --- | --- | --- | --- | --- | --- | --- | --- | --- | --- | --- | --- | --- | --- | --- | --- | --- | --- | --- | --- | --- | --- | --- | --- | --- | --- | --- | --- | --- | --- | --- | --- | --- | --- | --- | --- | --- | --- | --- | --- | --- | --- | --- | --- | --- | --- | --- | --- | --- | --- | --- | --- | --- | --- | --- | --- | --- | --- | --- | --- | --- | --- | --- | --- | --- | --- | --- | --- | --- | --- | --- | --- | --- | --- | --- | --- | --- | --- | --- | --- | --- | --- | --- | --- | --- | --- | --- | --- | --- | --- | --- | --- | --- | --- | --- | --- | --- | --- | --- | --- | --- | --- | --- | --- | --- | --- | --- | --- | --- | --- | --- | --- | --- | --- | --- | --- | --- | --- | --- | --- | --- | --- | --- | --- | --- | --- | --- | --- | --- | --- | --- | --- | --- | --- | --- | --- | --- | --- | --- | --- | --- | --- | --- | --- | --- | --- | --- | --- | --- | --- | --- | --- | --- | --- | --- | --- | --- | --- | --- | --- | --- | --- | --- | --- | --- | --- | --- | --- | --- | --- | --- | --- | --- | --- | --- | --- | --- | --- | --- | --- | --- | --- | --- | --- | --- | --- | --- | --- | --- | --- | --- | --- | --- | --- | --- | --- | --- | --- | --- | --- | --- | --- | --- | --- | --- | --- | --- | --- | --- | --- | --- | --- | --- | --- | --- | --- | --- | --- | --- | --- | --- | --- | --- | --- | --- | --- | --- | --- | --- | --- | --- | --- | --- | --- | --- | --- | --- | --- | --- | --- | --- | --- | --- | --- | --- | --- | --- | --- | --- | --- | --- | --- | --- | --- | --- | --- | --- | --- | --- | --- | --- | --- | --- | --- | --- | --- | --- | --- | --- | --- | --- | --- | --- | --- | --- | --- | --- | --- | --- | --- | --- | --- | --- | --- | --- | --- | --- | --- | --- | --- | --- | --- | --- | --- | --- | --- | --- | --- | --- | --- | --- | --- | --- | --- | --- | --- | --- | --- | --- | --- | --- | --- | --- | --- | --- | --- | --- | --- | --- | --- | --- | --- | --- |
